# Growth performance, carcass characteristics, fatty acid profile, and meat quality of male goat kids supplemented by alternative feed resources: bitter vetch and sorghum grains

**DOI:** 10.5194/aab-67-481-2024

**Published:** 2024-10-07

**Authors:** Soumaya Boukrouh, Ali Noutfia, Nassim Moula, Claire Avril, Julien Louvieaux, Jean-Luc Hornick, Jean-François Cabaraux, Mouad Chentouf

**Affiliations:** 1 Department of Veterinary Management of Animal Resources, FARAH Center, Faculty of Veterinary Medicine, University of Liège, Liège, Belgium; 2 Regional Center of Agricultural Research of Tangier, National Institute of Agricultural Research, Rabat, Morocco; 3 AgroBiosciences et Chimie, Haute École Provinciale de Hainaut Condorcet, Ath, Belgium

## Abstract

Bitter vetch and sorghum grains are alternative local feed resources that are underutilized in the southern Mediterranean area. This study aimed to assess the effects of incorporating these grains into the diet of local goat breeds on growth performance, carcass characteristics, and meat quality. Twenty-four goat kids were divided into three groups. The control group received a conventional diet consisting of oat hay, barley, and fava beans. In the first group, fava beans were replaced with bitter vetch, and in the second group, barley was replaced with sorghum. At the end of the trial, the animals were slaughtered and carcass characteristics and meat fatty acid profiles of the longissimus dorsi (LD) muscle were determined. Alternative grain incorporation had no significant effect on the growth parameters. Still, it significantly affected carcass characteristics, especially in the sorghum group compared to the control group, where mesenteric fat was lower (266 vs. 437 g). The back color was lighter ( $L^{*}=55.1$ vs. 59.1) and less yellow ( $a^{*}=-1.29$ vs. 2.22). The diet also influenced the meat's chemical composition, with less protein and ash in the chevrons of animals receiving bitter vetch and sorghum grains, respectively. Regarding the fatty acid (FA) profile, sorghum grains had decreased C18:2 n-6 and polyunsaturated FA (PUFA), whereas bitter vetch grains had increased C18:3 n-3, elongase activity, and nutritive value index (NVI). The control group exhibited intermediate results for C15:0, C16:0, C16:1, C20:3 n-3, n-3, health promoting index (HPI), and thrombogenic index (TI). No significant effects were reported for saturated FA (SFA) and monounsaturated FA (MUFA). Bitter vetch and sorghum grains can be safely incorporated into fattening diets of goat kids.

## 1 Introduction

Goat (*Capra hircus*) breeding has been a prevalent occupation of many people in northern Morocco since ancient times. It plays an important socioeconomic role by providing food and contributing to more than 70 % of the income of rural mountain communities. Goat herds are mainly made up of local indigenous populations that are well adapted to their environment. Some female goats are used for herd replacement, whereas the remaining animals are used for meat consumption (Godber et al., 2020).

Nowadays, consumers are becoming more nutrition- and health-conscious. Interest in lean chevon meat with low cholesterol and saturated fatty acids has increased. Lean meat has been shown to have fewer negative health effects (Mazhangara et al., 2019). Chevon meat has a favorable chemical nutrient composition, with comparable arginine, isoleucine, lysine, methionine, threonine, and tryptophan concentrations in beef, pork, and lamb (Mazhangara et al., 2019). Goat meat is a major source of micronutrients, particularly iron, potassium, and vitamin B12. These essential elements have been reported to prevent anemia, which is a threat to women of childbearing age, particularly in rural areas (Mwangi et al., 2017). It is also an important source of magnesium and potassium (Osman and Mahgoub, 2012), which are important for enzymatic reactions in the body, energy metabolism (Fiorentini et al., 2021), maintaining healthy blood pressure, and functioning of the nervous system (Stone and Weaver, 2021).

Goat breeding is conducted using the traditional silvopastoral and agro-silvopastoral systems. However, these areas have undergone serious degradation and biodiversity decline (Chebli et al., 2021b). Due to climate change, farmers tend to reduce the size of herds during drought periods to a level that can be sustainably fed. This reduction also helps alleviate the pressure on degraded rangelands (Godber et al., 2020). Therefore, there is a growing need for new resources to support goat production and to mitigate rangeland degradation.

Bitter vetch (*Vicia ervilia*) and sorghum (*Sorghum bicolor* (L.) Moench) are underused crops in northern Morocco (Boukrouh et al., 2023b, 2024; Hmimsa and Ater, 2008). Bitter vetch, an ancient legume native to the Mediterranean region, is cultivated as both fodder and grain. These grains are primarily used as a protein source (270 g kg DM ${}^{-1}$ , with DM being dry matter) (Sadeghi et al., 2009). This interesting grain nutritive value guaranteed the continuous cultivation of *Vicia ervilia* as ruminant feed in Morocco, Spain, and Türkiye. However, the presence of antinutritional factors limits the amount of bitter vetch that can be incorporated into the diet of non-ruminants because of its low utilization efficiency (Sadeghi et al., 2004). The effect of incorporating bitter vetch grains on the growth and meat quality of poultry and sheep has been well documented (Abdullah et al., 2010; Sadeghi et al., 2009).

Corn grains are a staple in ruminant diets because of their high starch content (Gómez et al., 2016). Although corn grains share some nutritive similarities with sorghum, sorghum generally contains more fiber and less starch than corn (Johnston and Moreau, 2017). Sorghum grains tend to have higher fat, protein, and mineral content such as calcium and iron (Tuna and Bressani, 1992). *Sorghum bicolor* is particularly noted for its resource use efficiency, exhibiting higher drought resistance, adaptability to different environments and soil conditions, and low fertilizer requirements (Assefa et al., 2013; Safian et al., 2022).

To the best of our knowledge, the use of bitter vetch and sorghum grains in the diet of goat kids has not been extensively studied. Promising results from such studies could reinforce the use of these local plants as alternative feed concentrates, potentially leading to more sustainable goat farming practices. This would help to decrease goat pressure on silvopastoral rangelands and allow the use of plants that are more resilient to drought and climate change. This study aimed to analyze the effects of incorporating bitter vetch and sorghum grains on the performance, carcass characteristics, and meat quality of goat kids.

## 2 Materials and methods

### 2.1 Experimental animals and diets

The study was conducted at the Bougdour experimental station (35°67 ${}^{\prime }$ N, 5°85 ${}^{\prime }$ W) at Tangier's Regional Agricultural Research Center (INRA; Tangier, Morocco). The sample size (number of goats) was estimated using the G*Power 3.1.4.9. Assuming a statistical power of 0.95 and a significance level of p<0.05 , 24 goat kids were required to compose the sample size to expect a statistically significant difference according to an effect size close to 0.25.

Goat kids from the local Beni Arouss breed were divided into three homogeneous groups based on initial body weight ( 9.43±0.1 kg) and age ( 86.5±12.5 d). Each group was housed in a single pen with a metal barrier to separate the experimental groups. Thermal conditions and humidity were meticulously controlled. The groups were randomly allocated to one of the three treatments (Table 1). The control group (Co; n=8 ) received a conventional diet typically used by regional farmers for fattening, consisting of oat hay, barley, and fava beans. The experimental groups were fed modified diets: one with bitter vetch substituting fava beans (BV; n=8 ) and the other with sorghum replacing barley (SRG; n=8 ). The forage-to-concentrate ratio is 50:50 .

**Table 1:** Diet composition.

	Co	BV	SRG
Diet ingredients (on a DM basis)			
Oat hay (g kg DM ${}^{-1}$ )	491	492	496
Bitter vetch grains (g kg DM ${}^{-1}$ )	0	298	0
Sorghum grains (g kg DM ${}^{-1}$ )	0	0	190
Barley grains (g kg DM ${}^{-1}$ )	186	192	0
Fava bean grains (g kg DM ${}^{-1}$ )	312	0	300
Urea (g kg DM ${}^{-1}$ )	4.4	11.6	7.4
Vitamin–mineral supplement ${}^{*}$	6.6	6.4	6.6
(g kg DM ${}^{-1}$ )			

Co: control diet, BV: diet with bitter vetch replacing fava bean, SRG: diet with sorghum replacing barley. ${}^{*}$ Per kg vitamin–mineral supplement: 2310 IU (international unit) retinol, 528 IU cholecalciferol, 9.9 mg α -tocopherol, 26.4 mg manganese, 33 mg zinc, 1.58 g calcium, and 330 mg magnesium.

The diets were isoenergetic and isonitrogenous and were formulated specifically to meet the requirements of growing goat kids. The kids were weaned at approximately 2.5–3 months of age and underwent a 15 d adaptation period prior to the start of the 90 d experimental period. All the feedstuffs were purchased from a local market. To formulate the diets accurately, a representative sample of each feedstuff was collected at the beginning of the experiment and each diet was sampled twice a week for the first 3 weeks of the trial. The chemical compositions of the ingredients and diets are presented in Tables 1, 2, and 3. Oat hay was finely chopped using a forage chopper equipped with a 2.5 cm screen and manually mixed with the concentrate in the feeder. The kids were collectively fed. The animals had access to fresh and clean water ad libitum. The total mixed ration was provided ad libitum twice daily, at 08:00 and 18:00 LT. The offered rations were adapted to allow for 10 % feed refusal to ensure that the kids were not underfed. All experiments and analytical methods were performed in accordance with the relevant regulations and guidelines of the National Center of Agricultural Research (license no. 01/CRRAT/2017).

**Table 2:** Chemical composition of diets.

	Co	BV	SRG
Nutrient composition			
Dry matter (g kg ${}^{-1}$ )	913	907	912
Ash (g kg DM ${}^{-1}$ )	63.7	53.2	60.1
Protein (g kg DM ${}^{-1}$ )	160	160	160
Ether extract (g kg DM ${}^{-1}$ )	19.7	15.7	21.2
Neutral detergent fiber (g kg DM ${}^{-1}$ )	477	453	457
Acid detergent fiber (g kg DM ${}^{-1}$ )	264	267	249
Acid detergent lignin (g kg DM ${}^{-1}$ )	46.0	36.2	43.0
Crude fiber (g kg DM ${}^{-1}$ )	215	195	202
Nitrogen-free extract (g kg DM ${}^{-1}$ )	651	631	650
In vitro enzymatic organic matter digestibility (g kg DM ${}^{-1}$ )	671	689	642
Metabolizable energy (ME; MJ kg DM ${}^{-1}$ )	8.52	8.42	8.18
Forage unit for meat (FUMeat kg DM ${}^{-1}$ )	0.70	0.70	0.71
PDIE (g kg DM ${}^{-1}$ )	80.0	75.9	81.5
RuProBal	29.2	22.7	29.8
Total phenols (g kg DM ${}^{-1}$ )	5.21	3.86	12.44
Condensed tannins (g kg DM ${}^{-1}$ )	1.71	0.61	1.57
Hydrolyzable tannins (g kg DM ${}^{-1}$ )	0.60	0.31	0.59
Urea (g kg DM ${}^{-1}$ )	4.27	11.7	7.43
Fatty acid profile of the diet (g 100 g FA ${}^{-1}$ )			
(calculated from feedstuff fatty acid profile)			
C12:0	0.000	0.292	0.074
C14:0	3.76	3.77	2.85
C14:1	0.000	0.249	0.000
C16:0	42.2	41.8	37.8
C16:1	0.710	0.679	1.13
C15:0	0.000	0.124	0.000
C17:0	0.952	1.02	0.797
C18:0	10.3	12.4	8.75
C18:1 n-9	8.63	7.44	14.7
C18:2 n-6	14.7	12.2	19.0
C18:3 n-3	16.4	16.6	12.2
C18:3 n-6	0.000	0.013	0.031
9t-C18:1	0.091	0.127	0.811
C20:0	1.126	0.638	0.753
C20:1	0.587	1.92	0.584
C22:0	0.604	0.480	0.221
C23:0	0.000	0.150	0.254

Co: control diet, BV: diet with bitter vetch replacing fava bean, SRG: diet with sorghum replacing barley, PDIE: digestible proteins in the intestines allowed by energy, RuProBal: rumen protein balance.

### 2.2 Chemical analysis

All feed/diet samples were analyzed at the INRA-Tangier laboratory using AOAC (1990) methods (Tables 2 and 3). Samples were first dried at 55 °C until they reached a constant weight and then ground and sieved through a 1 mm mesh. Dry matter was obtained by drying 100 g of each sample at 105±1 °C to a constant weight (method 934.01). The ash content was measured by incinerating 2 g of the sample in a muffle furnace at 550 °C for 12 h (method 942.05). Ether extract (EE) was extracted using a Soxhlet apparatus, with diethyl ether as the solvent (method 963.15). Nitrogen was quantified using the Kjeldahl method (mineralization, distillation, and titration) (method 977.02). The crude protein (CP) content was calculated by multiplying the nitrogen content by 6.25. The acid detergent lignin (ADL), acid detergent fiber (ADF), neutral detergent fiber (NDF), and crude fiber (CF) contents were analyzed using an ANKOM 200 Fiber Analyzer (ANKOM Technology, Macedon, NY, USA) following the method of Van Soest et al. (1991) for ADL, ADF, and NDF and method 962.09 for CF. The NDF was determined using α -amylase and sodium sulfite. The nitrogen-free extract (NFE) content was estimated according to the following equation, Eq. (1):

1 $$\mathrm{NFE}\;\;({g\;\mathrm{kg}\;\mathrm{DM}}^{-1})=100-(\mathrm{EE}+\mathrm{CP}+\mathrm{CF}+\mathrm{ash}).$$

In vitro enzymatic dry matter (IVDMD) and organic matter (IVOMD) digestibility were determined using the method described by Aufrère and Michalet-Doreau (1983). The metabolizable energy (ME) of the experimental diets was estimated based on the IVDMD (%) according to AOAC (1990) using Eq. (2):

2 $$\mathrm{ME}\;\;(\mathrm{MJ}\;\mathrm{kg}\;{\mathrm{DM}}^{-1})=0.17\times \mathrm{IVDMD}-2.$$

In vitro enzymatic CP degradability was determined as described by Aufrère and Cartailler (1988) to calculate the digestible proteins in the intestines allowed by nitrogen (PDIN) and the digestible proteins in the intestines allowed by energy (PDIE). The forage units for meat production (FUMeat; 1 FUMeat =1700 kcal or 7.12 MJ), PDIN, and PDIE were calculated using the INRAtion^®^ software (INRAE, Paris, France). The rumen protein balance (RuProBal) was calculated using the following equation, Eq. (3):

3 RuProBal=(PDIN-PDIE)/0.64.

Total phenols (TPs) and tannins (TTs) were quantified according to procedures described by Makkar et al. (1993). Condensed tannins (CTs) were assayed using the method described by Porter et al. (1985). The fatty acid (FA) profile of the feedstuff was extracted using sulfuric acid and determined using gas chromatography as described by O'Fallon et al. (2007). The FA profiles of the feedstuffs were used to calculate the FA profiles of the three diets. The detected data are listed in Table 3.

**Table 3:** Chemical composition of dietary ingredients.

Items	Oat hay	Bitter vetch	Fava bean	Sorghum	Barley
		grains	grains	grains	grains
Dry matter (g kg ${}^{-1}$ )	899.9	907.7	904.6	906.9	902.4
Crude protein (g kg DM ${}^{-1}$ )	62.1	234.0	262.6	88.7	111.8
Ether extract (g kg DM ${}^{-1}$ )	11.8	10.9	19.7	40.7	31.9
Ash (g kg DM ${}^{-1}$ )	77.1	31.0	49.0	13.1	24.7
Neutral detergent fiber (g kg DM ${}^{-1}$ )	571.4	160.2	141.5	110.2	181.4
Acid detergent fiber (g kg DM ${}^{-1}$ )	432.9	74.9	107.5	60.8	87.9
Acid detergent lignin (g kg DM ${}^{-1}$ )	61.2	8.59	26.7	6.70	17.6
Crude fiber (g kg DM ${}^{-1}$ )	324.0	44.1	65.3	17.7	57.6
Nitrogen-free extract (g kg DM ${}^{-1}$ )	525.0	680.0	603.5	839.8	774.0
In vitro enzymatic organic matter digestibility (g kg DM ${}^{-1}$ )	483.3	892.9	695.7	753.9	825.7
Metabolizable energy (MJ kg DM ${}^{-1}$ )	5.4	11.2	9.7	10.3	11.0
Forage unit for meat (FUMeat kg DM ${}^{-1}$ )	0.48	0.92	0.78	0.91	0.95
PDIE (g kg DM ${}^{-1}$ )	74.0	115.0	71.0	85.0	67.0
RuProBal	-46.9	57.8	132.8	-40.6	-12.5
Total phenols (g kg DM ${}^{-1}$ )	6.42	1.46	5.43	37.1	0.92
Hydrolyzable tannins (g kg DM ${}^{-1}$ )	0.44	0.26	1.09	0.11	0.09
Condensed tannins (g kg DM ${}^{-1}$ )	0.41	1.06	4.07	0.38	0.71

PDIE: digestible proteins in the intestines allowed by energy, RuProBal: rumen protein balance.

Slaughter procedures and carcass trait measurements. At the beginning and end of the experiment, body weights of the kids were recorded to determine their initial and final weights. Average daily gain (ADG) was calculated based on the initial and final body weight differences divided by the number of days in the trial period (90 d). Following a 24 h fasting period with unrestricted access to water, the animals were weighed to establish their slaughter body weight (SBW) and then slaughtered according to the guidelines of the ethical standards of the Regional Center of Agricultural Research of Tangier (license no. 01/CRRAT/2017). Immediately after slaughter and the removal of non-carcass components, anterior and posterior paws, head, fleece, pluck (trachea, lungs, trachea, heart, pancreas, liver, kidneys, testes), gastrointestinal tract (GIT), perirenal and mesenteric fat, hot carcass weight (HCW) were determined. Non-carcass components were weighed, and the GIT was weighed and empty after hand washing.

Dressing percentage (DP) was determined as the ratio of HCW to SBW. After the carcasses were chilled for 24 h at 4 °C, cold carcass weight (CCW) was recorded. A pH meter was used to measure rumen pH directly after GIT removal (Hanna HI98120, Hanna Instruments). The carcass length (CL), thigh length (TL), thigh width, thigh thickness (TT), shoulder perimeter, shoulder length, rib cage length, and width were measured. The compactness index (CI) and muscle index (MI) were calculated based on the CCW-to-CL and TL-to-TT ratios, respectively. The conformation index was calculated as the sum of CI and MI.

The evaluation of color parameters including lightness ( $L^{*}$ ), redness ( $a^{*}$ ), and yellowness ( $b^{*}$ ) was carried out 24 h post mortem in different regions of the carcass, including the belly, back, saddle, and tail outline. The $L^{*}$ value indicates luminosity ( $L^{*}=0$ being black and 100 white), the $a^{*}$ value ranges from green ( - ) to red ( + ), and the $b^{*}$ value extends from blue ( - ) to yellow ( + ). The reading was performed with a Konica Minolta CR-400 colorimeter, with a measurement and illumination area of Ø 8 and Ø 11 mm, respectively, and a 0° viewing angle, calibrated using a white CR-A43 calibration plate. Measurements were performed three times for each region, and the average of triplicate measurements was considered for each animal.

A piece of longissimus dorsi (LD) muscle from each carcass was excised 24 h after slaughter at the mid-point. The samples were then split into two sections. The first was directly used to determine the physical characteristics, and the second was ground for homogenization to determine the chemical composition. The pH of the muscles was determined both directly on the hot carcass and 24 h post mortem using a penetration pH meter (Hanna HI98120, Hanna Instruments). The color parameters, $L^{*}$ , $a^{*}$ , and $b^{*}$ , were also determined for the meat samples. The hue angle and chroma indices were determined using $a^{*}$ and $b^{*}$ indices according to King et al. (2012) using Eqs. (4) and (5) as follows:

4chroma=(a*2+b*2)0.5,5hue angle=arctangent(b*/a*)×[360/(2×3.14)]).

The water-holding capacity of the muscles was determined by cutting each sample into 2 g cubes, which were then placed on a filter paper sandwiched between two acrylic plates and subjected to a load of 10 kg for 5 min (Kauffman et al., 1986). The samples were weighed, and water loss was quantified as the percentage difference in weight before and after load exposure. Meat tenderness was determined in both muscles according to the method described by Sarriés and Beriain (2006). Six pieces, each measuring 1.27 cm per side and 2.00 cm in height, aligned parallel to the muscle fibers, were excised from each sample using a cork borer. The three pieces were placed in plastic bags, hermetically sealed, and cooked in a water bath at 75 °C for 40 min. At the end of cooking, the internal temperature of the meat reached 70 °C. The cooked samples were refrigerated for 24 h. The tenderness of both raw (24 h post mortem) and cooked (48 h post mortem) samples was measured using the Warner–Bratzler shear force protocol. This was performed using a TA.HDplusC texture analyzer with a V-shaped cutting blade (Stable Micro Systems Ltd., Godalming, Surrey, UK). Each sample was sheared perpendicular to the direction of the muscle fibers, and the force required was recorded in newtons (N).

The chemical composition of the meat was determined according to AOAC (1990). Initially, ground meat samples were thawed in a refrigerator for 12 h at 4 °C. To determine the moisture content, the meat samples were placed in an oven at 105 °C for 24 h, and the weight loss from drying provided the moisture percentage. The ash content was determined by adding 1 mL of magnesium acetate to the sample, which is followed by incineration for 5 h at 550 °C in a muffle furnace. The difference between the initial and ashed weights represents the ash percentage. The meat fat content was determined by acid hydrolysis; 5 g of the meat sample was boiled in 100 mL of 3N hydrochloric acid for 1 h. The resulting mixture was filtered, and fat was extracted using a Soxhlet apparatus. Protein content was calculated using the Kjeldahl method, where the total nitrogen content was multiplied by 6.25 to obtain the protein percentage. Fatty acid analysis was performed as previously described by Folch et al. (1957). The observed FAs were grouped into different categories (Adeyemi et al., 2015; Boukrouh et al., 2023a; Chen and Liu, 2020).

### 2.3 Statistical analysis

The experimental data were processed using the SAS software (version 9.4; SAS Institute Inc., Cary, NC, USA). The effects of diet were tested using a linear mixed model for growth performance, slaughter, carcass measurements, meat chemical composition, and FA profile, including the random effects of goats, according to Eq. (6):

6 $$Y_{ij}=\mu +D_{i}+G_{ij}+e_{ijk},$$

where $Y_{ij}$ is the dependent variable, μ is the mean, $D_{i}$ is the fixed effect of the i th modality of diet (Co, BV, or SRG), $G_{ij}$ is the random effect of the goat, and $e_{ijk}$ is the residual error. To compare the means of the test groups with those of the control group, Tukey's test was performed when a significant effect of the model was observed ( p<0.05 ).

## 3 Results and discussion

### 3.1 Animal performance and, slaughter and carcass characteristics

Growth parameters including initial, final, and slaughter body weight; average daily gain; hot and cold carcass weight; and dressing percentage were not statistically different between the three groups (9.4 kg, 15.5 kg, 15.0 kg, 67.6 g d ${}^{-1}$ , 6.8 kg, 6.5 kg, and 44.6 %, respectively) (Table 4). Diets had very little effect on non-carcass components. Although the full and empty GIT weights were similar among the three groups, the rate of empty GIT was higher in the SRG group. The proportion and weight of the pluck, anterior and posterior paws, and head were similar, but the fleece rate was lower in the BV and SRG diets than in the Co group. Methionine and cysteine are the primary limiting factors for bitter vetch grains (Sadeghi et al., 2009). In addition, sorghum grains contain less methionine than barley grains (Al-Marzooqi, 2020). Cysteine and methionine are the limiting amino acids in keratin synthesis and are among the limiting amino acids in meat production (Cao et al., 2021). Moreover, these limiting AA are prioritized for muscle growth rather than tegument growth. The differences in the AA content of the diets could explain the differences observed in the fleece rate (lowest in the SRG and BV groups) and protein content of the muscles (lowest in the two BV muscles). Several factors influence the carcass composition of goats, such as the concentrate level and type, forage type, and protein level, and source (Pophiwa et al., 2020). The SRG diet significantly reduced mesenteric fat weight but did not affect perirenal weight and rate. Carcass measurements (carcass length, thigh length, width, and thickness; shoulder length; and rib cage length and width) did not vary according to the diet. The carcass compactness index is an important economic indicator because the meat market prefers more compact carcasses (Nascimento et al., 2018). The distributed diet did not affect this parameter. However, the lower muscle and conformity indices of goat kids receiving BV or sorghum grains could negatively affect their incorporation and, thus, their use. However, the present muscle and conformity indices were higher than those reported by Lahkim Bennani et al. (2022) and El Otmani et al. (2021b) for kids of the Beni Arouss breed. The incorporation of bitter vetch and sorghum grains into the diets significantly affected carcass color. The backs of BV and SRG goat-kid carcasses were lighter ( $L^{*}$ ) than those of the control group ( p<0.05 ). Fat deposition and its distribution within muscle tissues can affect light scattering, thereby influencing the $L^{*}$ value. The SRG diet, which tended to reduce mesenteric fat, may also impact subcutaneous fat, contributing to a lighter appearance of the carcass. Indeed, the yellowness ( $b^{*}$ ) of the back, which is positively correlated with fat (Dunne et al., 2006), was lower in the SRG compared to Co diet.

**Table 4:** Growth, rumen pH, and carcass characteristics of goat kids according to their diet.

		Co	BV	SRG	SEM	p value
Initial body weight (kg)		10.3	9.0	9.0	2.610	0.610
Final body weight (kg)		17.1	15.1	14.3	3.385	0.310
Average daily gain (g d ${}^{-1}$ )		76.1	68.3	58.5	3.848	0.191
Slaughter body weight (kg)		16.5	14.6	13.8	3.703	0.319
Hot carcass weight (kg)		7.6	6.8	5.9	1.838	0.230
Dressing percentage (%)		46.1	45.5	42.3	3.091	0.321
Cold carcass weight (kg)		7.4	6.5	5.6	1.783	0.224
Gastrointestinal tract						
Weight of full GIT (g)		3567	3646	3684	290.6	0.963
Weight of empty GIT (g)		567	467	726	228.4	0.100
Rate of empty GIT (%)		3.34 ${}^{b}$	3.07 ${}^{b}$	5.05 ${}^{a}$	0.346	0.025
Rumen pH		5.55 ${}^{b}$	6.08 ${}^{\mathrm{ab}}$	6.18 ${}^{a}$	0.11	0.025
Fleece						
Weight (g)		1249	998	917	254.3	0.070
Rate (%)		7.28 ${}^{a}$	6.53 ${}^{b}$	6.46 ${}^{b}$	0.423	0.004
Pluck						
Weight (g)		863	809	729	170.5	0.354
Rate (%)		5.08	5.32	5.21	0.618	0.769
Anterior paws						
Weight (g)		323	258	265	62.6	0.148
Rate (%)		1.91	1.70	1.87	0.243	0.238
Posterior paws						
Weight (g)		257	217	188	52.2	0.072
Rate (%)		1.52	1.43	1.31	0.149	0.054
Head						
Weight (g)		1299	1082	1021	232.3	0.100
Rate (%)		7.68	7.12	7.31	0.917	0.532
Perirenal fat (g)						
Weight (g)		234	218	172	63.1	0.176
Rate (%)		1.42	1.45	1.24	0.471	0.650
Mesenteric fat						
Weight (g)		437 ${}^{a}$	425 ${}^{a}$	266 ${}^{b}$	138.9	0.048
Rate (%)		2.53	2.78	1.90	1.621	0.089
Carcass length (cm)		50.0	47.2	46.9	4.287	0.376
Thigh length (cm)		26.1	27.8	26.1	1.921	0.182
Tight width (cm)		11.7	10.4	10.1	1.157	0.060
Tight thickness (cm)		23.4	21.9	20.8	2.566	0.198
Shoulder length (cm)		25.7	24.1	23.9	1.656	0.132
Rib cage length (cm)		27.7	26.2	24.6	2.485	0.101
Rib cage width (cm)		15.6	15.8	16.1	1.362	0.860
Compactness index (g cm ${}^{-1}$ )		14.6	13.7	11.9	3.069	0.238
Muscle index		44.7 ${}^{a}$	37.6 ${}^{b}$	39.0 ${}^{\mathrm{ab}}$	0.043	0.018
Conformity index (g cm ${}^{-1}$ )		59.4 ${}^{a}$	51.3 ${}^{\mathrm{ab}}$	50.9 ${}^{b}$	0.061	0.036

**Table 4:** Continued.

		Co	BV	SRG	SEM	p value
Color parameters						
Belly	$L^{*}$	56.5	58.8	58.9	3.530	0.382
	$a^{*}$	13.7	13.3	12.6	2.936	0.774
	$b^{*}$	3.95	4.46	2.87	2.741	0.508
Back	$L^{*}$	51.3 ${}^{b}$	59.1 ${}^{a}$	55.1 ${}^{a}$	4.290	0.012
	$a^{*}$	9.74 ${}^{\mathrm{ab}}$	8.81 ${}^{b}$	11.4 ${}^{a}$	1.682	0.022
	$b^{*}$	2.22 ${}^{a}$	0.723 ${}^{\mathrm{ab}}$	$-1.29^{b}$	2.324	0.034
Tail outline	$L^{*}$	50.0	50.6	52.1	4.851	0.713
	$a^{*}$	18.3	20.0	18.3	3.102	0.469
	$b^{*}$	6.73	7.95	6.37	2.994	0.558

Co: control diet; BV: diet with bitter vetch replacing fava bean; SRG: diet with sorghum replacing barley; Pluck: liver, lung, pancreas, heart, spleen, and trachea; $L^{*}$ : lightness; $a^{*}$ : redness; $b^{*}$ : yellowness. ${}^{a,b,c}$ Values followed by different letters in the same row differ statistically according to Tukey's test at p<0.05 .

**Table 5:** Physical characteristics and chemical composition of the longissimus dorsi muscle of goat kids according to diet.

Physical characteristics	Co	BV	SRG	SEM	p value
pH 0	7.05	7.08	7.07	0.052	0.223
pH 24	6.06	6.03	6.05	0.062	0.086
Color indexes					
Lightness ( $L^{*}$ )	46.8	48.9	52.6	6.327	0.239
Redness ( $a^{*}$ )	21.65	22.8	18.84	3.331	0.073
Yellowness ( $b^{*}$ )	6.31	6.68	4.72	1.957	0.137
Hue angle (H°)	16.1	15.9	13.3	4.548	0.406
Chroma	7.45	7.65	6.74	0.725	0.054
Water loss (g kg ${}^{-1}$ )	147	174	169	13.6	0.209
Raw meat shear force (N)	109	101	102	22.7	0.853
Cooked meat shear force (N)	66.2	67.6	66.5	9.82	0.967
Chemical composition (g kg DM ${}^{-1}$ )					
Moisture	748.5	773.0	753.2	44.4	0.465
Ash	16.1 ${}^{a}$	15.5 ${}^{a}$	14.4 ${}^{b}$	0.916	0.010
Protein	220 ${}^{a}$	202 ${}^{b}$	215 ${}^{a}$	12.7	0.040
Fat	19.9	20.1	20.3	3.301	0.971

Co: control diet, BV: diet with bitter vetch replacing fava bean, SRG: diet with sorghum replacing barley, $L^{*}$ : lightness, $a^{*}$ : redness index, $b^{*}$ : yellowness index. ${}^{a,b,c}$ Values within the muscle followed by different letters in the same row differ significantly, according to Tukey's test at p<0.05 .

### 3.2 Meat quality

The physical characteristics and chemical composition of the meat are presented in Table 5. No significant differences between the diets were observed in the physical characteristics of the longissimus dorsi muscle. Regarding chemical composition, lower ash content was observed in the SRG group than in the Co one (14.4 vs. 16.1 g kg ${}^{-1}$ ), whereas reduced protein content was noted in the BV group (202 vs. 216 g kg ${}^{-1}$ ). Meat quality is generally affected by several factors, including breed, sex, age, and pre- and post-slaughter management practices (Pophiwa et al., 2020). Several antinutritional components, including protease inhibitors and canavanine, confer bitter taste or negative metabolic effects on bitter vetch grains (Sadeghi et al., 2009). Canavanine is a non-protein AA analogous to arginine that leads to the production of non-functional proteins (Sadeghi et al., 2009). Notably, the meat protein content in the BV group was lower than that in SRG and Co groups. This decrease in protein content could be related to the higher incorporation level of bitter vetch grains (30 %). In lamb diets, bitter vetch was incorporated only at 15 % and showed no difference in meat protein compared to Co. The observed ADGs were higher than the 35 g d ${}^{-1}$ reported by El Otmani et al. (2021) for animals of the same breed, whereas the final body weight, hot and cold carcass weights, and dressing percentage were in the range reported by the same author. The mean pH 0 h and 24 h after slaughter were higher than the normal range of 5.6–5.8 reported for LD, which are acceptable values for goat meat commercialization (Gawat et al., 2023). The post-slaughter decline in pH is a crucial step in the transformation of muscles into meat. Typically, an elevated ultimate pH results in tougher, darker, and more perishable meat. This could be attributed to pre-slaughter stress, which causes a decrease in glycogen levels and a subsequent reduction in lactic acid concentrations (Rojas et al., 2022). Meat color is the first criterion used by consumers to judge the meat quality. Red color is influenced by the level and state of myoglobin (Zhu et al., 2022). Despite the variation in back carcass color, meat color was not significantly different between the groups, probably due to variations in fat accumulation in carcasses. Moreover, Vioque et al. (2020) reported that phenols exhibit antioxidant properties that indirectly prolong the red color stability of meat by delaying the oxidation of metmyoglobin (Zhu et al., 2022). The absence of differences in meat color may be due to low phenol concentration. Indeed, despite the highest total phenol content observed in the SRG diet, the total phenol content in the three diets was lower than 20–30 g kg DM ${}^{-1}$ , which has been reported to affect meat antioxidative stability (García et al., 2019). Moreover, as $L^{*}$ values were higher than 37–40.4, the meat in the following experiment was not considered dark, firm, or dry (Muchenje et al., 2009).

Tenderness is the most important organoleptic trait contributing to consumer acceptance and eating satisfaction with meat (Garmyn, 2020). The shear force test is generally used to measure meat tenderness and is positively affected by collagen content (Florek et al., 2022). The absence of differences between diets for shear force values in the two muscles was expected because of the low differences in the ultimate pH between diets. The tenderness of the cooked LD (61.8 N) was in the range of values reported in the literature (47–94 N) (Cao et al., 2021; Hwang and Joo, 2017; Mwangi et al., 2017). The test diets were supplemented with urea to make them isoproteic. However, BV meat had a lower protein content, which is possibly due to the lower content of bitter vetch in essential and limiting amino acids (methionine and cysteine) (Sadeghi et al., 2009), as explained above, and could also be due to the presence of antinutritional factors, including canavanine. In addition, the meat protein results were close to 23 % DM reported by Mazhangara et al. (2019) for chevons.

### 3.3 Meat fatty acid profile

The incorporation of bitter vetch and sorghum grains significantly affected the FA profile, families, ratios, and indices of longissimus dorsi meat FA (Table 6). The SRG diet was associated with significantly lower PUFA and PUFA/SFA values compared with the Co diet, which was related to lower C18:2 n-6. Conversely, the BV diet increased C18:3 n-3, elongase activity and nutritive value index (NVI). The SRG diet had lower C15:0, C20:3 n-3, and n-3 and higher C16:0, C16:1, and TI compared to the BV diet. The fatty acid profile has a crucial impact on meat quality (Mazhangara et al., 2019). The lower levels of odd-chain FA (C15:0) in the SRG diet can be attributed to the slower fermentability of sorghum starch compared to that of barley starch. Highly fermentable starch-based diets have been reported to promote the proliferation of ruminal amylolytic bacteria, which promotes propionate, the precursor for odd-chain FAs production (Vlaeminck et al., 2006). As in the BV diet, barley starch is the main source of energy, and its higher and rapid fermentability has probably led to reduced rumen pH and inhibition of biohydrogenation, which could explain the higher C14:1 and C16:1.

The diets did not affect MUFAs and SFAs, but it is interesting to consider the family of PUFAs frequently represented in the feed of vegetal origin – that is, C18:2 n-6 and C18:3 n-3 – because they are important for improving meat quality in ruminants. The lower levels of these FAs in the LD muscle of the SRG group and consequently the lower PUFA / SFA ratio are in line with the lower amounts of C18:3 n-3 measured in the corresponding diet (Table 3) and because of the biohydrogenation that reduced C18:2 n-6 despite its higher concentration in the SRG diet. The incorporation of bitter vetch and sorghum grains did not affect the n-6 / n-3 ratio. The desirable range for this index is 4:1 to maintain a balanced and healthy life (Ponnampalam et al., 2021). Bitter vetch grain incorporation potentially increased elongase activity and the nutritive value index. Recommendations to consume food with a reduced TI and high NVI have also been suggested to improve human health (Chen and Liu, 2020). The NVI of 3.31 in the current study was higher than the values reported for goats, whereas a TI of 1.14 was at an intermediate level (Taboada et al., 2022; Yang et al., 2022).

**Table 6:** Fatty acid profile and summaries (g 100 g FA ${}^{-1}$ ), ratios, and indices of the longissimus dorsi muscle of goat kids according to diet.

	Co	BV	SRG	SEM	p value
C4:0	0.42	0.47	0.51	0.163	0.596
C6:0	0.37	0.38	0.51	0.135	0.119
C8:0	0.38	0.47	0.40	0.153	0.509
C10:0	0.17	0.28	0.23	0.096	0.120
C11:0	0.06	0.06	0.07	0.027	0.063
C12:0	0.23	0.15	0.21	0.066	0.054
C13:0	0.51	0.61	0.61	0.142	0.404
C14:0	1.23	0.93	1.04	0.280	0.157
C15:0	4.61 ${}^{\mathrm{ab}}$	5.53 ${}^{a}$	3.63 ${}^{b}$	1.06	0.007
C16:0	13.0 ${}^{\mathrm{ab}}$	11.6 ${}^{b}$	14.3 ${}^{a}$	1.38	0.004
C17:0	3.27	3.22	2.13	1.01	0.070
C18:0	17.5	18.3	18.0	1.44	0.579
C20:0	0.50	0.57	0.54	0.217	0.830
C21:0	0.37	0.46	0.45	0.159	0.519
C22:0	1.25	1.24	1.22	0.097	0.883
C23:0	5.74	5.65	6.24	1.09	0.527
C24:0	2.10	2.42	2.48	0.274	0.045
SFA	51.6	52.4	52.6	2.04	0.675
C14:1	0.26	0.21	0.21	0.057	0.227
C15:1	1.25	1.08	0.86	0.327	0.111
C16:1	1.35 ${}^{\mathrm{ab}}$	0.95 ${}^{b}$	1.66 ${}^{a}$	0.355	0.003
C17:1	1.00	0.96	0.74	0.200	0.049
9t-C18:1	1.03	1.05	1.13	0.268	0.759
C18:1 n-9	27.5	27.5	27.5	2.22	0.996
C20:1	0.56	0.63	0.83	0.262	0.164
C22:1 n-9	0.39	0.39	0.40	0.058	0.923
C24:1	1.15	1.26	1.33	0.198	0.266
MUFA	34.5	34.0	34.7	2.35	0.817
6t-C18:2	1.73	1.44	1.69	0.185	0.488
C18:2 n-6	7.30 ${}^{a}$	6.99 ${}^{\mathrm{ab}}$	6.26 ${}^{b}$	0.751	0.046
C18:3 n-3	0.34 ${}^{b}$	0.43 ${}^{a}$	0.22 ${}^{b}$	0.072	<0.001
C18:3 n-6	0.26	0.27	0.25	0.001	0.262
C20:2	0.61	0.59	0.56	0.154	0.852
C20:3 n-3	0.58 ${}^{\mathrm{ab}}$	0.70 ${}^{a}$	0.35 ${}^{b}$	0.173	0.002
C20:3 n-6	0.44	0.59	0.60	0.132	0.074
C20:4 n-6	0.45	0.50	0.49	0.058	0.260
C20:5 n-3	1.31	1.40	1.43	0.197	0.550
C22:2	0.49	0.41	0.50	0.218	0.703
C22:6 n-3	0.32	0.36	0.34	0.215	0.947
PUFA	13.8 ${}^{a}$	13.7 ${}^{\mathrm{ab}}$	12.7 ${}^{b}$	0.818	0.030
DFA	65.8	66.0	65.4	1.42	0.747
n-3	2.56 ${}^{\mathrm{ab}}$	2.88 ${}^{a}$	2.34 ${}^{b}$	0.349	0.020
n-6	10.2	9.79	9.29	0.872	0.190
n-9	29.0	28.9	29.1	2.181	0.986
EPA + DHA	1.64	1.76	1.77	0.337	0.726
PUFA / SFA	0.27 ${}^{a}$	0.26 ${}^{\mathrm{ab}}$	0.24 ${}^{b}$	0.017	0.025
MUFA / PUFA	2.51	2.50	2.74	0.284	0.195
EPA / AA	2.97	2.83	2.96	0.387	0.738
DHA / AA	0.73	0.72	0.71	0.402	0.998
n-6 / n-3	3.99	3.48	4.03	0.581	0.147
Δ9 C16	0.10	0.08	0.11	0.008	0.083
Δ9 C18	0.62	0.61	0.61	0.033	0.787
AA/EPA + DHA	0.66	0.72	0.69	0.213	0.860
Elongase activity	0.75 ${}^{b}$	0.79 ${}^{a}$	0.74 ${}^{b}$	0.022	0.002
TI	1.05 ${}^{\mathrm{ab}}$	1.00 ${}^{b}$	1.14 ${}^{a}$	0.094	0.025
NVI	3.57 ${}^{b}$	4.07 ${}^{a}$	3.31 ${}^{b}$	0.462	0.012

Co: control diet; BV: diet with bitter vetch replacing fava bean; SRG: diet with sorghum replacing barley. MUFA: monounsaturated fatty acid; PUFA: polyunsaturated fatty acid; SFA: saturated fatty acid; DFA: desirable fatty acid; EPA: eicosapentaenoic acid (20:5 n-3); DHA: docosahexaenoic acid (C22:6 n-3); AA: arachidonic acid (C20:4 n-6); LA: linoleic acid (C18:2 n-6); ALA: α -linolenic acid (C18:3 n-3); Δ 9C16: (C16:1)/(C16:1 + C16:0) activity of Δ 9 desaturase enzyme to convert C16:0 into C16:1; Δ 9C18: (C18:1)/(C18:1 + C18:0) activity of Δ 9 desaturase enzyme to convert C18:0 to C18:1; TI: thrombogenic index: C14:0 + C16:0 + C18:0)/[( 0.5×Σ MUFA) + (0.5 × Σ n-6 PUFA) + ( 3×Σ n-3 PUFA) + (n-3/n-6)]; NVI: nutritive value index: (C18:0 + C18:1 n-9 + 9t-C18:0)/C16:0; elongase activity: (C18:1 n-9 + C18:0)/(C18:1 n-9 + C18:0 + C16:1 + C16:0). ${}^{a,b,c}$ Values within muscle followed by different letters in the same row differ statistically by Tukey's test at p<0.05 .

## 4 Conclusions

Bitter vetch and sorghum grains are two alternative energy and protein resources that can be incorporated into the diet of goat kids without negatively impacting carcass and growth parameters. However, meat quality, including fatty acid profiles, varied according to the diet. Further investigations are recommended to evaluate the effects of bitter vetch and sorghum grains on ruminal microbiota and goat milk quality. Different levels of grain incorporation and treatments should also be evaluated.

## Data Availability

The datasets presented in this study are available upon request from the corresponding author.

## Code availability

The software code presented in this study is available upon request from the corresponding author.

## References

[bib1.bib1] Abdullah AY, Muwalla MM, Qudsieh RI, Titi HH (2010). Effect of bitter vetch (Vicia ervilia) seeds as a replacement protein source of soybean meal on performance and carcass characteristics of finishing Awassi lambs. Trop Anim Health Pro.

[bib1.bib2] Adeyemi KD, Ebrahimi M, Samsudin AA, Sabow AB, Sazili AQ (2015). Carcass traits, meat yield and fatty acid composition of adipose tissues and Supraspinatus muscle in goats fed blend of canola oil and palm oil. J Anim Sci Technol.

[bib1.bib3] Al-Marzooqi W (2020). Comparative Amino Acid Ileal Digestibility of Feed Ingredients Measured with Indigenous and Commercial Strains of Chickens. Int J Poult Sci.

[bib1.bib4] AOAC (Association of Official Analytical Chemists) (1990). Official Methods of Analysis, 15th ed..

[bib1.bib5] Assefa Y, Roozeboom K, Thompson C, Schlegel A, Stone L, Lingenfelser JE (2014). Corn and Grain Sorghum Comparison.

[bib1.bib6] Aufrère J, Cartailler D (1988). Mise au point d'une méthode de laboratoire de prévision de la dégradabilité des protéines alimentaires des aliments concentrés dans le rumen. Ann Zootech.

[bib1.bib7] Aufrère J, Michalet-Doreau B, Boucque CV (1983). In vivo digestibility and prediction of digestibility of some by-products. Agriculture: Feeding Value of By-Products and Their Use by Beef Cattle, Proceedings of an EEC Seminar.

[bib1.bib8] Boukrouh S, Noutfia A, Moula N, Avril C, Hornick JL, Chentouf M, Cabaraux JF (2023). Effects of Sulla Flexuosa Hay as Alternative Feed Resource on Goat's Milk Production and Quality. Animals.

[bib1.bib9] Boukrouh S, Noutfia A, Moula N, Avril C, Louvieaux J, Hornick JL, Cabaraux JF, Chentouf M (2023). Ecological, Morpho-Agronomical, and Bromatological Assessment of Sorghum Ecotypes in Northern Morocco. Sci Rep.

[bib1.bib10] Boukrouh S, Noutfia A, Moula N, Avril C, Louvieaux J, Hornick JL, Chentouf M, Cabaraux JF (2024). Characterisation of bitter vetch ecotypes: an ancient and promising legume. Exp Agr.

[bib1.bib11] Cao Y, Yao J, Sun X, Liu S, Martin GB (2021). Amino acids in the nutrition and production of sheep and goats. Adv Exp Med Biol.

[bib1.bib12] Chebli Y, El Otmani S, Elame F, Moula N, Chentouf M, Hornick JL, Cabaraux JF (2021). Silvopastoral System in Morocco: Focus on Their Importance, Strategic Functions, and Recent Changes in the Mediterranean Side. Sustainability.

[bib1.bib13] Chen J, Liu H (2020). Nutritional Indices for Assessing Fatty Acids: A Mini-Review. Int J Mol Sci.

[bib1.bib14] Dunne PG, O'mara FP, Monahan FJ, Moloney AP (2006). Changes in colour characteristics and pigmentation of subcutaneous adipose tissue and M. longissimus dorsi of heifers fed grass, grass silage or concentrate-based diets,. Meat Sci.

[bib1.bib15] El Otmani S, Chebli Y, Hornick J-L, Cabaraux J-F, Chentouf M (2021). Growth performance, carcass characteristics and meat quality of male goat kids supplemented by alternative feed resources: Olive cake and cactus cladodes. Anim Feed Sci Tech.

[bib1.bib16] Fiorentini D, Cappadone C, Farruggia G, Prata C (2021). Magnesium: Biochemistry, Nutrition, Detection, and Social Impact of Diseases Linked to Its Deficiency. Nutrients.

[bib1.bib17] Florek M, Domaradzki P, Skałecki P, Ryszkowska-siwko M, Ziomek M, Tajchman K, Gondek M, Pyz-Łukasik R (2022). Content and Solubility of Collagen and Their Relation to Proximate Composition and Shear Force of Meat from Different Anatomical Location in Carcass of European Beaver (Castor fiber). Foods.

[bib1.bib18] Folch J, Lees M, Stanley GHS (1957). A simple method for the isolation and purification of total lipids from animal tissues. J Biol Chem.

[bib1.bib19] García EM, López A, Zimerman M, Hernández O, Arroquy JI, Nazareno MA (2019). Enhanced oxidative stability of meat by including tannin-rich leaves of woody plants in goat diet, Asian-Australas. J Anim Sci.

[bib1.bib20] Garmyn A (2020). Consumer Preferences and Acceptance of Meat Products. Foods.

[bib1.bib21] Gawat M, Boland M, Singh J, Kaur L (2023). Goat Meat Production and Quality Attributes. Foods.

[bib1.bib22] Godber OF, Chentouf M, Wall R (2020). Sustainable goat production: modelling optimal performance in extensive systems. Anim Prod Sci.

[bib1.bib23] Gómez LM, Posada SL, Olivera M, Gómez LM, Posada SL, Olivera M (2016). Starch in ruminant diets: A review. Rev Colomb Cienc Pec.

[bib1.bib24] Hmimsa Y, Ater M (2008). Agrodiversity in the Traditional Agrosystems of the Rif Mountains (North of Morocco). Biodiversity.

[bib1.bib25] Hwang YH, Joo ST (2017). Fatty Acid Profiles, Meat Quality, and Sensory Palatability of Grain-fed and Grass-fed Beef from Hanwoo, American, and Australian Crossbred Cattle. Korean J Food Sci An.

[bib1.bib26] Johnston DJ, Moreau RA (2017). A comparison between corn and grain sorghum fermentation rates, Distillers Dried Grains with Solubles composition, and lipid profiles. Bioresource Technol.

[bib1.bib27] Kauffman RG, Eikelenboom G, Van der Wal PG, Merkus G, Zaar M (1986). The use of filter paper to estimate drip loss of porcine musculature. Meat Sci.

[bib1.bib28] King DA, Shackelford SD, Kalchayanand N, Wheeler TL (2012). Sampling and aging effects on beef longissimus color stability measurements. J Anim Sci.

[bib1.bib29] Lahkim Bennani M, Aarab A, Jaber A, Acherkouk M, El Ayadi M (2022). Effect of yellow sweetclover (Meli-lotus officinalis) hay compared with Lucerne (Medicago sativa) hay on carcass characteristics and meat quality of male goat kids. J Adv Vet Anim Res.

[bib1.bib30] Makkar HPS, Blummel M, Borowy NK, Becker K (1993). Gravimetric determination of tannins and their correlations with chemical and protein precipitation methods. J Sci Food Agr.

[bib1.bib31] Mazhangara IR, Chivandi E, Mupangwa JF, Muchenje V (2019). The Potential of Goat Meat in the Red Meat Industry. Sustainability.

[bib1.bib32] Mwangi MN, Phiri KS, Abkari A, Gbané M, Bourdet-Sicard R, Braesco VA, Zimmermann M. B. PrenticeAM (2017). Iron for Africa–Report of an Expert Workshop. Nutrients.

[bib1.bib33] Muchenje V, Dzama K, Chimonyo M, Strydom PE, Raats JG (2009). Relationship between pre-slaughter stress responsiveness and beef quality in three cattle breeds. Meat Sci.

[bib1.bib34] Nascimento UFS, Santos GRA, Azevedo CS, Macedo FAF, Gonçalves TR, Bomfim LELM, Farias JS, Santos ADF (2018). Performance and carcass characteristics of lambs 1/2 Dorper 
+
 1/2 Santa Inês, slaughtered with different thicknesses of subcutaneous fat. Rev Bras de Saude e Prod Anim.

[bib1.bib35] O'Fallon J, Busboom J, Nelson M, Gaskins CT (2007). A direct method for fatty acid methyl ester synthesis: application to wet meat tissues, oils, and feedstuffs. J Anim Sci.

[bib1.bib36] Osman NHI, Mahgoub O (2012). Goat Meat Production and Quality.

[bib1.bib37] Ponnampalam EN, Sinclair AJ, Holman BWB (2021). The Sources, Synthesis and Biological Actions of Omega-3 and Omega-6 Fatty Acids in Red Meat: An Overview. Foods.

[bib1.bib38] Pophiwa P, Webb EC, Frylinck LA (2020). review of factors affecting goat meat quality and mitigating strategies. Small Ruminant Res.

[bib1.bib39] Porter LJ, Hrstich LN, Chan BG (1985). The conversion of procyanidins and prodelphinidins to cyanidin and delphinidin. Phytochemistry.

[bib1.bib40] Rojas HE, Contreras RL, Schnake FG, Marín PM, Huissier PGL, Castillo PB (2022). Factors Determining Meat Quality and Cold Preservation Methods to Extend Shelf Life. J Biol Sci.

[bib1.bib41] Sadeghi G, Samie A, Pourreza J, Rahman HR (2004). Canavanine content and toxicity of raw and treated bitter vetch (Vicia ervilia) seeds for broiler chicken. Int J Poult Sci.

[bib1.bib42] Sadeghi GH, Pourreza J, Samei A, Rahmani H (2009). Chemical composition and some anti-nutrient content of raw and processed bitter vetch (Vicia ervilia) seed for use as feeding stuff in poultry diet. Trop Anim Health Pro.

[bib1.bib43] Safian N, Naderi MR, Torabi M, Soleymani A, Salemi HR (2022). Corn (Zea mays L.) and sorghum (Sorghum bicolor (L.) Moench) yield and nutritional quality affected by drought stress. Biocatal Agric Biotechnol.

[bib1.bib44] Sarriés MV, Beriain MJ (2006). Colour and texture characteristics in meat of male and female foals. Meat Sci.

[bib1.bib45] Stone M, Weaver C, Murrell TS, Mikkelsen RL, Sulewski G, Norton L, Thompson ML (2021). Improving Human Nutrition: A Critical Objective for Potassium Recommendations for Agricultural Crops. Improving Potassium Recommendations for Agricultural Crops.

[bib1.bib46] Taboada N, Fernández Salom M, Córdoba A, González SN, López Alzogaray S, Van Nieuwenhove C (2022). Administration of selected probiotic mixture improves body weight gain and meat fatty acid composition of creole goats. Food Biosci.

[bib1.bib47] Tuna E, Bressani R (1992). Chemical composition of 11 varieties of sorghum (Sorghum vulgare) before and after popping the kernels. Arch Latinoam Nutr.

[bib1.bib48] Van Soest PJ, Robertson JB, Lewis BA (1991). Methods for Dietary Fiber, Neutral Detergent Fiber, and Nonstarch Polysaccharides in Relation to Animal Nutrition. J Dairy Sci.

[bib1.bib49] Vioque J, Girón-Calle J, Torres-Salas V, Elamine Y, Alaiz M (2020). Characterization of Vicia ervilia (bitter vetch) seed proteins, free amino acids, and polyphenols. J Food Biochem.

[bib1.bib50] Vlaeminck B, Fievez V, Tamminga S, Dewhurst RJ, Van Vuuren A, De Brabander D, Demeyer D (2006). Milk odd-and branched-chain fatty acids in relation to the rumen fermentation pattern. J Dairy Sci.

[bib1.bib51] Yang Y, Wang Y, Shan H, Zheng Y, Xuan Z, Hu J, Wei M, Wang Z, Liu Q, Li Z (2022). Novel Insights into the Differences in Nutrition Value, Gene Regulation and Network Organization between Muscles from Pasture-Fed and Barn-Fed Goats. Foods.

[bib1.bib52] Zhu W, Han M, Bu Y, Li X, Yi S, Xu Y, Li J (2022). Plant polyphenols regulating myoglobin oxidation and color stability in red meat and certain fish: A review. Crit Rev Food Sci.

